# Transpiration Rate of White Clover (*Trifolium repens* L.) Cultivars in Drying Soil

**DOI:** 10.3389/fpls.2021.595030

**Published:** 2021-03-17

**Authors:** Lucy Egan, Rainer Hofmann, Shirley Nichols, Jonathan Hadipurnomo, Valerio Hoyos-Villegas

**Affiliations:** ^1^AgResearch Lincoln Research Centre, Christchurch, New Zealand; ^2^Faculty of Agriculture and Life Sciences, Lincoln University, Lincoln, New Zealand; ^3^AgResearch Ruakura Research Centre, Hamilton, New Zealand; ^4^Faculty of Agricultural and Environmental Sciences, McGill University, Montreal, QC, Canada

**Keywords:** transpiration, normalized transpiration rate, fraction of transpirable soil water, white clover, drought tolerance, abiotic stress tolerance

## Abstract

Determining the performance of white clover cultivars under drought conditions is critical in dry climates. However, comparing the differences in cultivar performance requires equivalent soil water content for all plants, to reduce the water deficit threshold eliciting stomatal closure. In this study, the objective was to compare the rate of stomatal closure in eighty white clover cultivars in response to soil drying. Two glasshouse experiments were conducted, and the daily transpiration rate was measured by weighing each pot. The transpiration rate of the drought-stressed plants were normalized against the control plants to minimize effects from transpiration fluctuations and was recorded as the normalized transpiration rate (NTR). The daily soil water content was expressed as the fraction of transpirable soil water (FTSW). The FTSW threshold (FTSWc) was estimated after which the NTR decreases linearly. The FTSWc marks the critical point where the stomata start to close, and transpiration decreases linearly. The significant difference (*p <* 0.05) between the 10 cultivars with the highest and lowest FTSWc demonstrates the cultivars would perform better in short- or long-term droughts.

## Introduction

White clover is the most important pastoral legume in temperate regions of the world and is usually grown in companion with ryegrass (Caradus et al., 1989). The pasture mix of ryegrass and white clover is common in a variety of grazing systems, including sheep and beef, deer, and dairy. Globally, white clover is an attractive plant to have in pastoral systems due to the nitrogen fixation ability and the resulting role in sustainable farming systems. White clover is economically important to New Zealand and fixes approximately 1.57 million tons of nitrogen annually (Caradus et al., 1996). New Zealand has the highest export share of white clover globally (57.5%), exporting approximately 4500 tons of white clover seed annually (Rattray, 2005). In a more recent report by Nixon (2016), the estimated direct and dependent industry GDP total contribution from white and red clover was ∼$2.3billion. The domestic impact was ∼$1.5million and the export impact was ∼$6.3million. Pyke et al. (2004) used data from Nas (2003) and estimated that in 2002, New Zealand had a 38% share of the global white clover seed production market. Hampton et al. (2012) stated in 2010 and 2011, 3108, and 3745 tons of white clover seed were produced in New Zealand.

Globally, drought stress is one of the major limiting factors in white clover performance in pastoral systems. Climate change predictions show that there will be a global increase in temperature of 4.5–18°C over the next century; increasing drought periods globally (Fischlin et al., 2007; IPCC, 2013). With the expansion of farming into arid geographic areas, climate change and increased water restrictions, there has been more urgency to breed and utilize cultivars that can perform under drought stress (Jahufer et al., 2013).

There have been several studies investigating the response of white clover to drought conditions (Thomas, 1984; Annicchiarico and Piano, 2004; Bermejo et al., 2006; Li et al., 2013). Thomas (1984) showed that the competitiveness of white clover in a sward is reduced significantly in drought-stressed environments which impacts severely on farm production.

Recurring droughts in New Zealand have increased farm management challenges for farmers. There have been many studies aimed at increasing performance in drought through agronomic practices (van den Bosch et al., 1993; Widdup and Barrett, 2011). Although there has been little success in breeding drought tolerance white clover cultivars, differing grazing management schemes can aid in protecting the plants under drought conditions (Brock, 1988).

White clover cultivars need to be exposed to the same drought condition to make accurate assumptions on differences of phenotypic performance in drought (Tuberosa, 2012). In a field trial environment, phenotypic differences in performance can be due to differences in water regimes. Other measures of drought tolerance are needed to accurately determine the performance of plants in drought conditions (Sinclair and Ludlow, 1986; Weisz et al., 1994; Ray and Sinclair, 1997, 1998).

Selecting genotypes that are conservative in water use is a selection strategy to increase drought tolerance (Ray and Sinclair, 1997, 1998; Giday et al., 2013; Fanourakis et al., 2015). Traits such as normalized transpiration rate (NTR) and fraction of transpirable soil water (FTSW) can be used to determine cultivars or families that perform better in drought-like conditions (Lecoeur and Sinclair, 1996; Ray and Sinclair, 1997, 1998; Miller, 2000). Short-term drought is defined as being less than 6 months, while long-term drought is defined as longer than 6 months (Dziegielewski, 2003). Transpiration rate is controlled by stomatal closure (Ray and Sinclair, 1997, 1998). Genotypes that are more sensitive to drought will close their stomata earlier to preserve soil water content and may perform better in long-period drought conditions as water-conserving efforts occur earlier. Genotypes that have late stomatal closure may be better suited for short-period drought conditions.

Currently, there is limited published literature on calculating the NTR and FTSW critical threshold (FTSWc) of white clover cultivars in drying soil. This study aims to build on previous reports of FTSWc in plant species (Sinclair and Ludlow, 1986; Weisz et al., 1994; Lecoeur and Sinclair, 1996; Ray and Sinclair, 1998; Gholipoor et al., 2013). We used a panel of 80 white clover cultivars to determine the FTSWc, marking permanent stomatal closure and the start of senescence.

## Methodology

### Germplasm

The 80 cultivars used in this study were the same as in Hoyos-Villegas et al. (2019) and were released from 1920 to 2010 by both public and private breeding programs. The cultivars were from across New Zealand, Australia, United Kingdom, and the United States of America. The cultivars ranged in leaf size from small (*N* = 1), to medium (*N* = 53), and to large (*N* = 26) (Table 1).

**TABLE 1:** The cultivar names, the decade of release, countries of release, registered leaf sizes, dry weights of the drought and irrigated treatments and the critical fraction of transpirable soil water (FTSWc) threshold of eighty white clover cultivars.

Cultivar	Decade of release	Country of origin	Leaf size	Dry weight drought	Dry weight irrigated	FTSWc
LSD*_(0.05)_*				1.99	1.99	2.02
Dutch white	1920	Netherlands	Medium	13.58	17.75	0.23
Irrigation	1930	Australia	Medium	18.63	20.78	/
Kent White	1930	UK	Small	16.85	26.28	0.17
Louisiana	1930	USA	Medium	17.78	29.08	0.25
S 100	1930	UK	Medium	18.73	20.70	0.35
Kersey	1940	UK	Medium	21.55	28.68	/
S 184	1940	UK	Medium	17.18	23.05	0.28
California Ladino	1950	USA	Large	15.65	23.18	/
Grasslands Huia	1950	NZ	Medium	17.45	25.95	0.36
Ladino Gitante Lodigiano	1950	USA	Large	23.33	27.40	/
Louisiana S1	1950	USA	Medium	20.53	26.25	/
Pilgrim	1950	USA	Medium	17.35	29.25	0.28
Sonja	1950	Sweden	Large	18.70	23.30	0.33
Tribla	1950	Belgium	Medium	13.70	21.38	0.42
Clarence	1960	Australia	Medium	15.88	19.83	0.22
Crau	1960	France	Medium	16.43	22.38	/
Haifa	1960	Israel	Medium	14.95	22.35	0.38
Regal	1960	USA	Large	13.80	20.23	/
Donna	1970	UK	Medium	13.75	19.78	0.37
Lune de mai	1970	France	Large	17.28	24.23	0.21
Milkanova	1970	Denmark	Medium	17.18	24.13	0.26
Olwen	1970	UK	Large	15.08	21.88	/
Pitau	1970	NZ	Medium	14.00	19.20	0.48
Radi	1970	Poland	Large	15.85	24.23	0.23
Sacramento	1970	Poland	Large	15.85	21.48	0.49
Siral	1970	Australia	Medium	15.25	18.35	/
Alice	1980	UK	Medium	14.18	19.38	0.36
Aran	1980	Ireland	Large	13.30	22.78	/
Kopu	1980	NZ	Large	17.05	21.93	/
Lirepa	1980	Germany	Medium	17.65	22.83	0.34
Menna	1980	UK	Medium	12.23	21.90	0.39
Merwi	1980	Belgium	Medium	15.35	22.33	0.37
Osceola	1980	USA	Medium	12.93	17.20	0.42
Ross	1980	Ireland	Large	16.75	24.95	0.40
AberHerald	1990	UK	Medium	14.13	22.25	0.12
Challenge	1990	NZ	Medium	14.40	21.40	0.27
Crescendo Ladino	1990	USA	Large	12.88	19.75	0.32
Dacia	1990	Romania	Large	16.53	26.03	0.28
Jumbo	1990	USA	Medium	18.80	24.00	0.20
Kopu II	1990	NZ	Large	14.33	20.80	0.34
Le Bons	1990	NZ	Medium	16.10	18.80	0.24
Prop	1990	NZ	Medium	14.20	23.00	0.34
Regal Graze	1990	USA	Large	18.15	28.40	/
Reisling	1990	Netherlands	Medium	15.75	22.75	0.30
Sustain	1990	NZ	Medium	16.05	17.50	0.32
Triffid	1990	France	Large	16.23	24.30	0.30
Waverley	1990	Australia	Large	12.93	22.53	/
AberConcord	2000	UK	Medium	15.95	23.13	0.20
AberDance	2000	UK	Medium	17.88	22.65	0.35
AberNormous	2000	UK	Large	16.78	21.58	0.26
Aquiles	2000	Uruguay	Medium	20.50	24.40	0.13
Artigas	2000	Uruguay	Large	16.10	22.90	/
Barblanca	2000	France	Medium	15.75	25.08	0.27
Bounty	2000	NZ	Medium	12.53	20.43	0.47
Chieftain	2000	Ireland	Medium	17.25	21.40	0.17
Crusader	2000	France	Medium	15.60	16.40	/
Emerald	2000	NZ	Medium	15.98	20.78	0.33
Goliath	2000	Uruguay	Large	17.95	26.58	0.17
Klondike	2000	Denmark	Medium	13.95	25.33	/
Kotare	2000	NZ	Large	14.65	19.45	0.42
Quest	2000	NZ	Medium	13.95	20.95	0.33
Saracen	2000	Australia	Medium	15.28	24.20	0.27
Super Haifa	2000	Australia	Medium	15.93	21.75	0.24
Super Ladino	2000	Australia	Large	17.10	22.98	0.18
Tasman	2000	NE	Medium	13.48	21.80	0.30
Tillman II	2000	USA	Large	16.80	23.15	0.28
Tribute	2000	NZ	Medium	16.48	21.05	0.52
Trophy	2000	Australia	Medium	10.65	11.23	/
Vysocan	2000	Czech	Large	15.33	21.93	/
ABM21252	2010	NZ	Large	17.33	24.25	0.18
Calimero	2010	USA	Medium	17.93	22.50	/
Dairy B GC276	2010	Australia	Medium	16.18	21.58	/
Dairy D	2010	NZ	Medium	21.53	26.08	0.18
Elite Breeding A	2010	Australia	Medium	18.05	24.73	0.24
Kakariki	2010	NZ	Large	18.08	25.93	/
Katy	2010	USA	Medium	18.70	21.28	0.24
Legacy	2010	NZ	Large	18.15	27.98	0.29
Mainstay	2010	NZ	Medium	18.40	26.95	0.29
Quartz	2010	NZ	Medium	16.53	23.58	0.24
Weka	2010	NZ	Medium	18.75	25.98	0.18

**LSD_0.05_ is presented for the dry weights of the irrigated and drought cultivars and the FTSWc. The “/” denotes that the FTSW threshold was unable to be calculated for the cultivar because of the irregularities in the data for curve generation. Abbreviations: NZ, New Zealand; UK, United Kingdom; USA, United States of America.**

### NTR and FTSW Trial Design

**FIGURE 1:**
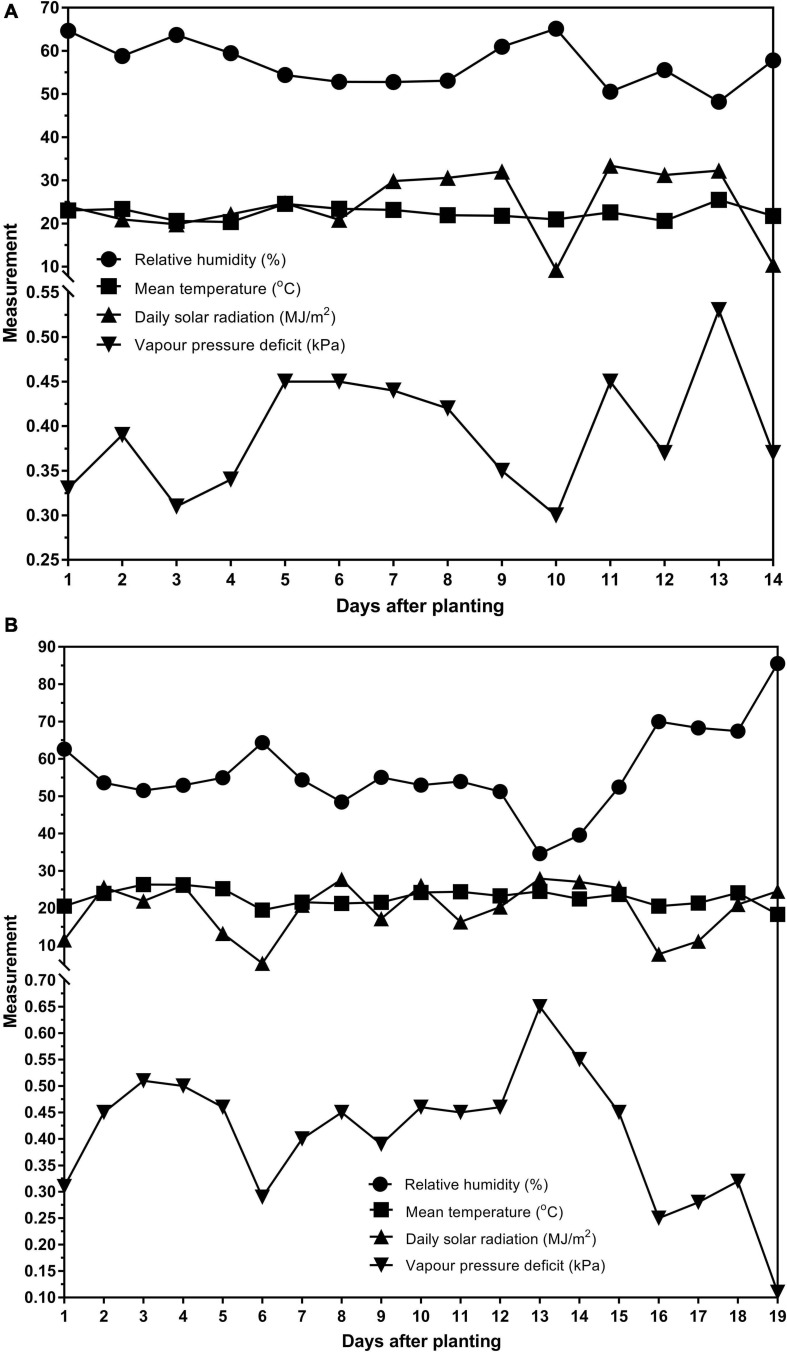
The relative humidity (%), mean temperature (°C), daily solar radiation (MJ/m^2^) and vapor pressure deficit (kPa) for the **(A)** 14 days of the first experiment (09/12/2016–22/12/2016) and **(B)** 19 days of the second experiment (02/02/2017–20/02/2017).

Two glasshouse experiments were conducted at AgResearch, Lincoln, New Zealand in the summer months (December–February). The data was from both trials were combined and analyzed together. The first experiment ran for 14 days (09/12/2016–22/12/2016) after planting. Weather and glasshouse data for that period showed that the average temperature was 22.39°C, the average relative humidity was 57.00% and the average total daily solar radiation was 24.39MJ/m^2^. The second experiment ran for 19 days (02/02/2017–20/02/2017) after planting. The average temperature was 22.79°C, the average relative humidity was 56.51% and the average total daily solar radiation was 19.82 MJ/m^2^. The average vapor pressure deficits for each experiment are reported in Figures 1A,B. The weather station was not equipped to measure sunshine hours.

The 80 cultivars were exposed to two treatments, irrigated (control) and drought, and replicated twice (technical replicates) in a randomized complete block design. Four stolons (biological replicates) were potted individually in potting mix in 4L pots and grown in a glasshouse with the optimum temperature of 20–25°C maintained. The pots were arranged in a 4 × 80 pot arrangement across 8 glasshouse tables. The plant stolons were cut and transplanted from trays and were young plants. Stolons from mother plants were cut after 3 weeks of growth. Vegetative growth is the primary way that white clover survives under natural conditions after it loses its taproot in the first year. We utilized stolons to emulate growth after the second year of growth under field conditions. The irrigated pots were watered with the exact amount of water that had transpired. Ten bare pots were used to measure water loss through evaporation. The pots were watered to container capacity and then left to drain and sealed on the bottom with duct tape. The saturated weight of each pot was recorded. Daily measurements of soil water content, measured as the weight of each pot, and the amount of water transpired, measured as the difference in pot weight, were recorded. The drought treatment ceased once the cultivars had died.

Dry weight was measured by the harvesting the roots and shoots together and weighed as total plant weight. The samples were dried at 80°C for 12 h and weighed (g) until a constant weight was reached. The leaf size used was the commercially stated leaf size associated with the cultivar.

### Calculations

Vapor pressure deficit (VPD) was calculated using the formula in Conaty et al. (2014):

$$V\,P\,D=e_{s}-e_{a}$$

where VPD is the difference between ambient water vapor (*e*_a_) and the saturated vapor pressure (*e*_s_) at the same temperature. The air temperature (*T*_a_ in °C) and relative humidity (RH in %) where:

$$e_{s}=0.6108\,e\,\left(\frac{17.27\,T_{a}}{T_{a}+237.3}\right)$$

$$e_{a}=\left(\frac{R\,H}{100}\right)\,e_{s}$$

The transpiration data were analyzed by the methodology described in Ray and Sinclair (1997).

The transpiration rate (TR) was calculated using the formula:

$$T\,R=\frac{W\,e\,i\,g\,h\,t\,o\,f\,d\,r\,o\,u\,g\,h\,t\,p\,o\,t}{W\,e\,i\,g\,h\,t\,o\,f\,c\,o\,n\,t\,r\,o\,l\,p\,o\,t}$$

where the soil water content and amount of water transpired were normalized against the control pots.

The NTR was calculated using the formula:

N⁢T⁢R=T⁢RD⁢a⁢y⁢ 3⁢a⁢n⁢d⁢ 5⁢a⁢v⁢e⁢r⁢a⁢g⁢e⁢T⁢R

The transpiration values were normalized against the days 3 and 5 average TR to minimize the effects of fluctuations in transpiration. The TR on days 3 and 5 are considered to be under well-watered conditions (Sinclair and Ludlow, 1986), allowing the plants to have an average NTR near-equal to one when sufficient soil water was available.

FTSW was calculated using the formula:

$$D\,a\,i\,l\,y\,F\,T\,S\,W=\frac{\left(D\,a\,i\,l\,y\,p\,o\,t\,w\,e\,i\,g\,h\,t-F\,i\,n\,a\,l\,p\,o\,t\,w\,e\,i\,g\,h\,t\right)}{\left(I\,n\,i\,t\,i\,a\,l\,p\,o\,t\,w\,e\,i\,g\,h\,t-F\,i\,n\,a\,l\,p\,o\,t\,w\,e\,i\,g\,h\,t\right)}$$

where the initial and final pot weights were the first and last day of the trials.

The relationship between the transpiration value and FTSW of each cultivar was explained by using non-linear regression to fit the equation:

$$N\,T\,R=\frac{1}{\left[1+A\times e\,x\,p\,\left(B\times F\,T\,S\,W\right)\right]}$$

Comparisons of the curve generated for each cultivar were based on 95% confidence intervals of coefficients *A* and *B*. *A* and *B* were empiric parameters generated through curve fitting. Plateau regression was used to determine the FTSWc. The curve predicts that NTR will remain near 1 up until a critical point, after which NTR decreases. The FTSWc is estimated, where NTR decreases linearly after that. The FTSWc marks the critical point where the stomata start to close, and transpiration decreases linearly.

### Statistical Analysis

ANOVA in GenStat (VSN-International, 2019) examined the main effects of water and cultivar, as well as their interaction. Before ANOVA, data were tested for homogeneity of variances and no transformation of the data was required.

A linear spline model was used to analyze the trend of the FTSWc over decades. Since the average trend showed a linear increase from 1920 up to 1960, then, changed into a decrease after 1960, the trend pattern was modeled as a linear spline model with a single knot at 1965. The linear spline trend model was estimated in regression by the formula:

I⁢n⁢f⁢l⁢e⁢c⁢t⁢i⁢o⁢n⁢p⁢o⁢i⁢n⁢t=a+b×T⁢i⁢m⁢e+c⁢x⁢T⁢i⁢m⁢e⁢_⁢2

where *Time* is a variable representing the decades in order, *Time_2* is a variable also representing decades in non-sequential order, and *a*, *b* and *c* are parameters to be estimated. In this formula, the increasing trend up to 1960 was estimated as a positive *b* value—i.e., *b* = increasing rate per decade till 1960, while the decreasing trend after 1960 was estimated as the sum of *b* + *c*, which should be a negative value—i.e., the absolute value of *b* + *c* = decreasing rate per decade after 1960. In addition, since the value of *c* indicated the difference of two trend slopes (one till 1960 and the other after 1960), the significance level (*p*-value) associated with *c* indicated if the change of the trend from the increase to the decrease was statistically significant.

Duncans lettering was used to indicate groups that are significantly different at the 5% significance level (Duncan, 1955).

## Results

### Dry Weights

**FIGURE 2:**
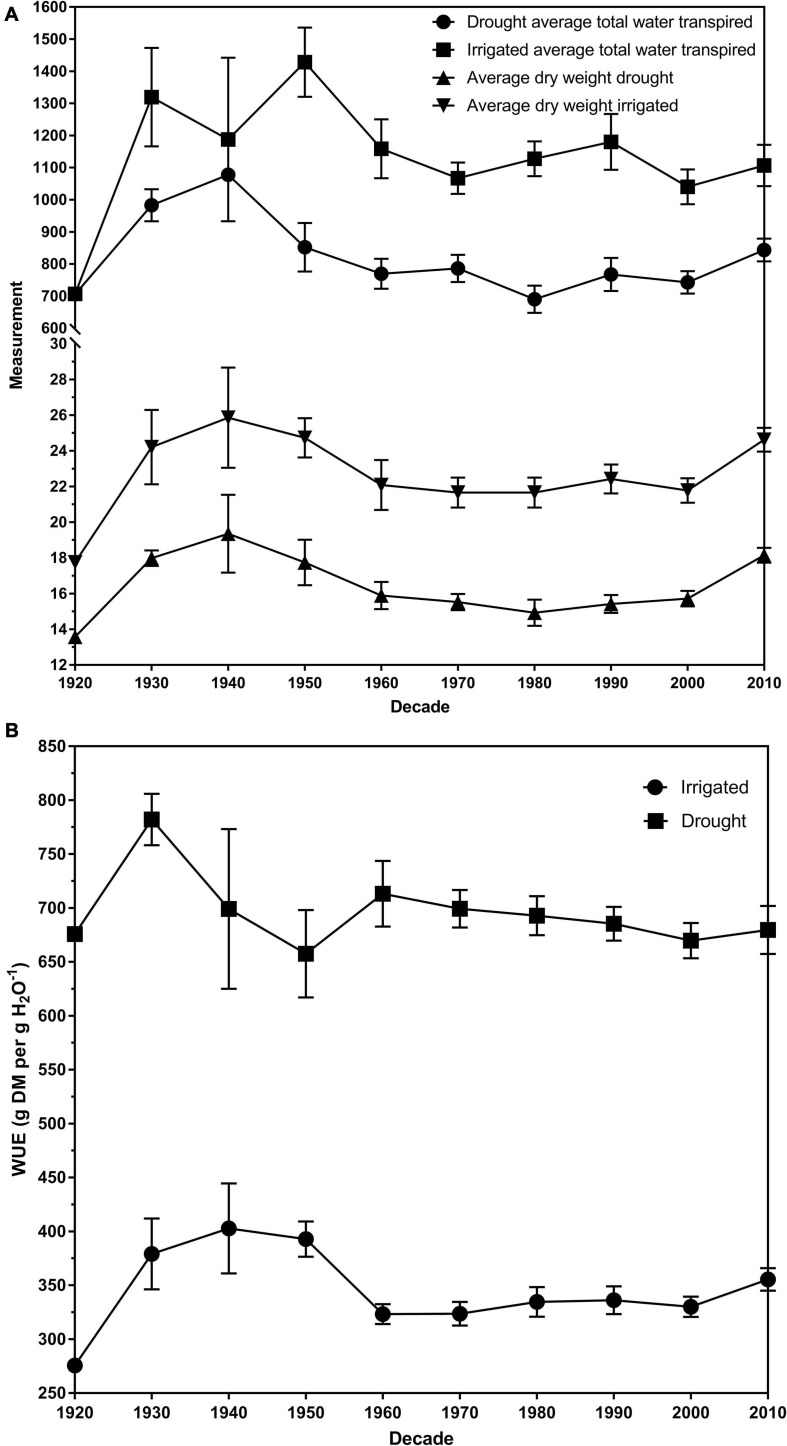
**(A)** The dry weights and transpiration rates for drought and irrigated plants for eighty white clover cultivars per decade of release. **(B)** The average water use efficiency (WUE) of drought and irrigated plants for eighty white clover cultivars per decade of release expressed as gram of dry matter per gram of water (g DM per g H_2_0^–1^). The error bars are the standard error of the means. All data was averaged across the two biological replicates.

The average total plant dry weight for drought and irrigated plants was 16.27 and 22.70 g, respectively. The plant total dry matter weights ranged from 11.23 to 29.25 g for control plants and 10.65 g and 23.32 for drought plants (Table 1). There was a significant overall difference (*p <* 0.01) in the dry weights of the cultivars when averaged across the two water treatments (Figures 2A,B; Duncan, 1955). The average dry weight of medium-leaved cultivars was highest between 1930 and 1950. The medium-leaved cultivars released in the 1920s were significantly different from all other groups for drought and irrigated plant dry weight (Supplementary Tables 1, 2). The large-leaved cultivars released in the 1950s and 1960s were significantly different from all other groups for drought plant dry weight (Supplementary Table 1). The cultivar and drought interaction was not significant.

Shoot to root ratio was unable to be calculated as only total plant weight was recorded. However, in future experiments the effect of drought on shoot to root ratio would be beneficial.

### Transpiration Rate, NTR, and FTSW

**FIGURE 3:**
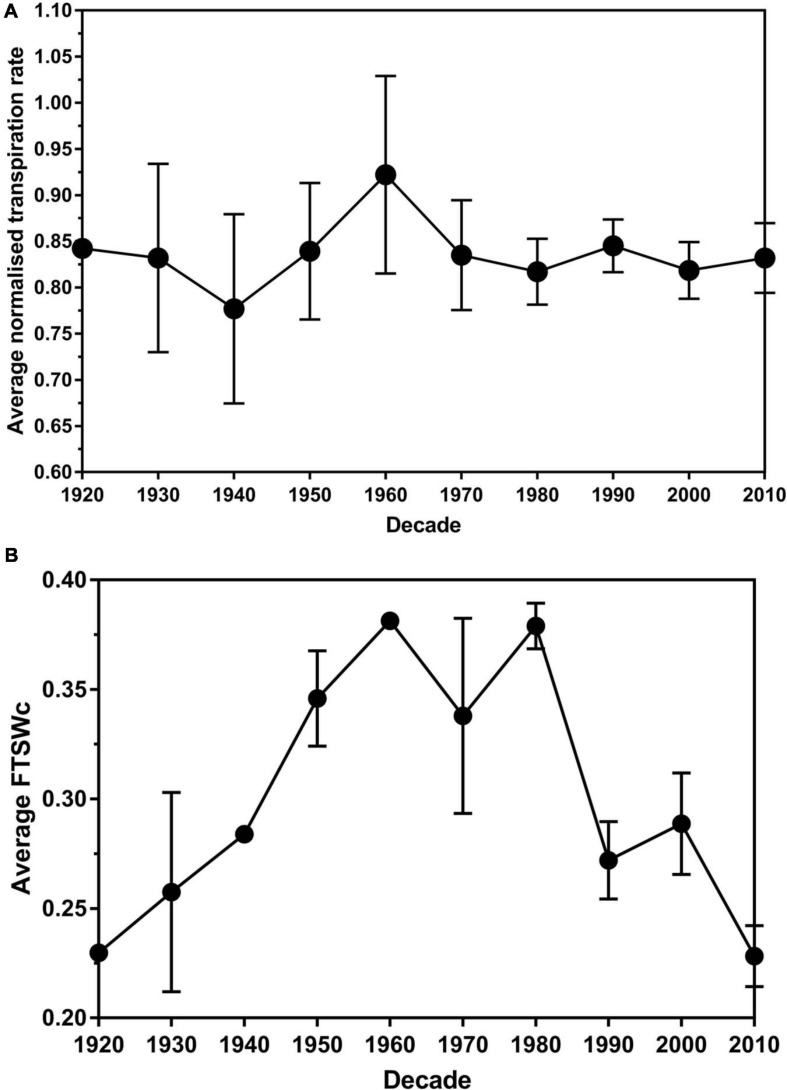
The average normalized transpiration rate **(A)** and critical fraction of transpirable soil water threshold (FTSWc) **(B)** per decade of release for eighty white clover cultivars. The error bars are the standard error of the means.

No significant difference (*P >* 0.05) in the average transpiration rate data between leaf size was found. There was a significant difference (*P <* 0.05) in the average transpiration rate data between the decade of cultivar release. Over the decades, the average NTR decreased from 1920 (0.84) to 1940 (0.78) before increasing to the peak in the 1960s (0.92) (Figure 3A). There was a decrease to 1980 (0.82), followed by a slight increase in the 1990s (0.84). The average NTR decreased in 2000 (0.81) before an increase in 2010 (0.83).

**FIGURE 4:**
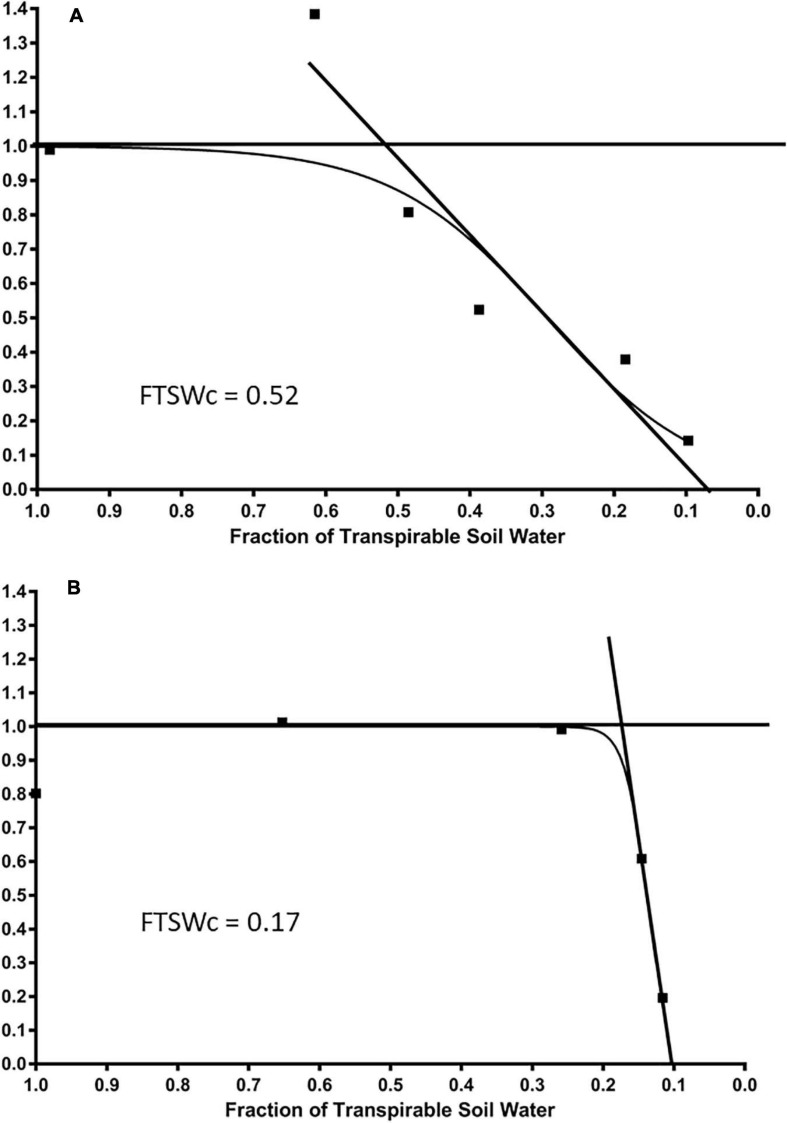
The average daily normalized transpiration rate (NTR) response to the fraction of transpirable soil water (FTSW) of two white clover cultivars, **(A)** Tribute, released in 2000, and **(B)** Chieftain, released in 2000, that show contrasting FTSWc. The FTSWc where NTR begins to decrease are shown.

A consistent relationship was found between NTR and FTSW values for each cultivar, as illustrated in Figure 4 by the cultivars Tribute and Chieftain (Figures 4A,B). On average among all cultivars, the NTR value was equivalent to well-watered plants until the FTSWc reached 0.29. The NTR value decreased linearly to 0 below an FTSW value of 0.29. Generally, there was no decrease in NTR until FTSW reached 0.7. The average number of days until the end of transpiration for the NTR was 9.95 and 14.48 for the FTSW. The FTSW values ranged from 0.11 to 0.50, with an average of 0.29.

### Stomatal Closure

Plateau regression was used to determine the FTSW value where the stomata permanently closed. The average FTSWc across the decades had an increasing trend from 1920 (0.23) to 1960 (0.38) (Figure 2B). There was a slight decrease in the 1970s (0.34) before it increased back to 0.38 in the 1980s. From the 1980s to 2010, there is a general decreasing trend until 2010 (0.23). The trend in the decade of release and FTSWc had an *r*^2^-value of 0.81. The *P*-value of 0.016 associated with parameter *b* indicated that the FTSWc value statistically significantly increased on average at a rate of 0.0329 (with a standard error of 0.013) per decade until 1960 (Supplementary Table 3). Then, the *P*-value of 0.002 associated with parameter *c* indicated that the increasing trend changed statistically significantly downwards after 1960. The *P*-value of 0.001 associated with the sum of parameters *b* + *c*, indicated that the FTSWc value statistically significantly decreased on average at a rate of 0.0307 (with standard error of 0.0089) per decade after 1960.

**FIGURE 5:**
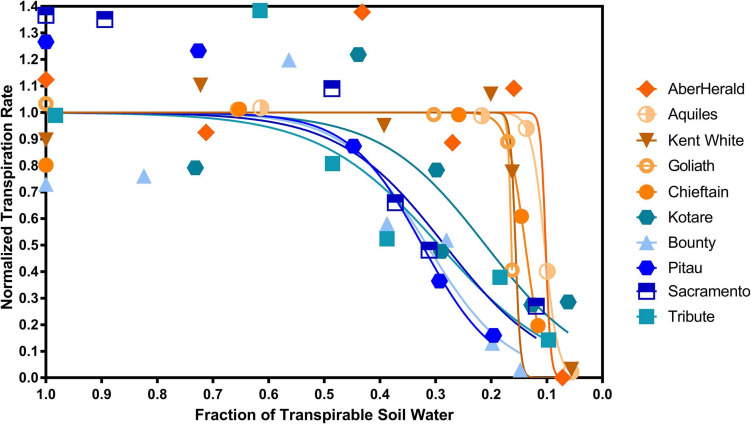
The five highest and five lowest fraction of transpirable soil water thresholds (FTSWc) for eighty white clover cultivars. The cultivars with the highest FTSWc (Kotare, Bounty, Pitau, Sacramento, and Tribute) are denoted in blue, while the cultivars with the lowest FTSW threshold (AberHerald, Aquiles, Kent White, Goliath, and Chieftain) are denoted in orange.

There was a significant (*P* < 0.001) difference between the 10 cultivars with the highest and lowest FTSWc (Figure 5). The five cultivars with the lowest FTSW were AberHerald (0.12), Aquiles (0.13), Kent White (0.17), Goliath (0.17), and Chieftain (0.17). The five cultivars with the highest FTSW were Kotare (0.44), Bounty (0.47), Pitau (0.48), Sacramento (0.49), and Tribute (0.52).

## Discussion

### FTSW Threshold

The objective of this study was to evaluate the response of 80 white cultivars to drying soil. The results of this experiment are similar to previous reports (Muchow and Sinclair, 1991). The transpiration rate in plant species has shown to be unaffected by drying soil until the FTSWc decreases to between approximately 0.25 and 0.35 (Ritchie, 1973; Meyer and Green, 1981; Sinclair and Ludlow, 1986; Rosenthal et al., 1987; Lecoeur and Sinclair, 1996; Ray and Sinclair, 1997, 1998; Miller, 2000; Gholipoor et al., 2013). The dependency of NTR and FTSW is shown through similar patterns of the NTR-FTSW relationship. The fact that the average FTSW in 1920 was similar to the average FTSW in 2010 suggests that breeding efforts have not implicated the relationship. Regardless of the decade of the release of the cultivar, the response to drought is similar.

White clover breeding in the 1900–1950s is documented most thoroughly in New Zealand compared to Europe and the United States (Zeven, 1991). The large majority of the understanding of white clover genetics and diversity and the effect of selection techniques began in the mid-1960s and onwards (Williams, 1987; Williams et al., 2007). Hoyos-Villegas et al. (2019) showed that breeding progress of white clover cultivars could be divided into two eras; pre- and post-1965. There were significant increases in white clover sward content and dry matter yield after 1965, but not pre-1965. The results in this study were consistent with the results found by Hoyos-Villegas et al. (2019) as the significant increase and decrease of FTSWc could be divided into the same two eras; pre- and-post 1965.

Prior to 1965, the breeding decisions for white clover breeding programs were based on increasing the performance of ecotypes and existing cultivars, and simple phenotypic selection. The breeding programs and trials were performed across multiple regions and trial sites, and populations were selected for broad adaptation across a range of farming systems (Williams et al., 2007). The breeding programs relied on the variation that was present within countries and local environments, as there was little germplasm exchange between countries. Cultivars bred post-1965 utilized foreign germplasm and selection techniques such as recurrent phenotypic selection and wide hybridization (Ellis and Young, 1967; Williams, 2014). In the 1980s in New Zealand, a large and stable clover seed export market had been established. The cultivars that were exported were bred through a variety of techniques. Local ecotypes and local populations were utilized for adaptation to the target environment, and elite breeding populations were incorporated for a range of desirable traits. The populations were combined and evaluated through phenotypic selection methods (Caradus and Christie, 1998). The introduction of mixed swards to evaluate populations and grazing animals as a selection pressure occurred after 1965 (Woodfield and Caradus, 1994). It is possible that the breeding objectives of production-based traits took priority and tied with the intensification of agriculture, reduced the drought tolerance of germplasm. Understanding the history of white clover breeding will inform future breeding decisions and increase efficiency of germplasm utilization.

Globally, germplasm exchange increased to widen the genetic base of populations in the later 1900s. Egan et al. (2019a, b) showed that in white and red clover, the introduction of foreign germplasm into the MFGC peaked in the 1970s and 1980s. By the 1980s, large-scale multi-country breeding programs were established (Williams et al., 2007). It is estimated that exotic germplasm used since the mid-1960s has contributed ∼$1 billion annually to the pastoral agricultural exports (Lancashire, 2006). The characterization of the germplasm and new methods of utilizing the foreign germplasm could be expected to increase the performance of white clover germplasm (Williams et al., 2007; Egan et al., 2019a).

However, although foreign germplasm has been utilized effectively worldwide (Rumball and Armstrong, 1974), the world checklists of white clover cultivars show that the large majority of the cultivars with good tolerance to drought and heat were released before 1965 (Caradus, 1986; Caradus and Woodfield, 1997). The rankings could suggest that locally adapted germplasm has outperformed foreign germplasm for drought tolerance. Conversely, germplasm collected from the Mediterranean has been used in breeding programs to produce cultivars with increased winter-growth activity (Cooper et al., 1997; Woodfield et al., 2001; Ayres et al., 2007).

Throughout the decades of breeding, different breeding goals have been the focus of breeding programs. Caradus et al. (1989) summarized the breeding goals for each decade of white clover breeding in New Zealand. The early programs focussed on advancing ecotypes and existing cultivars. In comparison, the later breeding programs focussed on whole plant production and the integration of different farm and grazing management practices. The only decade to focus specifically on physiological and morphological responses to environmental changes was the 1950s, where it is likely that drought tolerance was integrated into cultivars released. A focal breeding target in the 1970s was on the productivity. The lack of statistical significance difference between the average FTSW and the leaf size of cultivars suggests that all cultivars perform similarly under drought conditions. However, these results suggest that certain cultivars may be better utilized in certain environments and farming systems, i.e., short- and long-term droughts. Although the differences between the cultivars are not deemed statistically significant, the implications of a small difference in the NTR-FTSW relationship and FTSWc can be important knowledge for field conditions. Similarly, Barbour et al. (1996) assessed the water use efficiency of ten white clover cultivars under moisture stress and found that there was there were no significant differences between the cultivars. Although statistical significance was not found, the results are biologically relevant and could be utilized in different farming environments.

Understanding the relationship between drought tolerance and persistence will be beneficial for further development of cultivars (Sanderson et al., 2003). Persistence is defined as the maintenance of long-term agronomic yield and is a function of stolon growth and density (Williams, 1987; Annicchiarico and Piano, 2004). Drought is one of the main persistence-limiting traits (Bouton, 2012). Drought conditions limit white clover stolon branching and rooting (Chapman, 1983), and higher stolon density is often associated with superior water conservation (Collins, 1998). The success of a forage cultivar is largely dependent on the ability to survive summer droughts and retain productivity (Annicchiarico et al., 2011; Pecetti et al., 2011). Drought tolerance and stolon density can increase persistence. Hutchinson et al. (1995) carried out a 30 year study of environmental factors affecting the persistence of white clover and found that drought stress in late summer was the most critical limiting factor. Belaygue et al. (1996) saw an 80% decrease in stolon density when rainfall decreased by 30%. In orchardgrass, Saeidnia et al. (2018) noted that drought-tolerant genotypes had high persistence and Saeidnia et al. (2016) showed that drought conditions reduced forage yield and persistence.

Traditional breeding methods, such as phenotypic selection, have been the most common in white clover breeding programs (Hoyos-Villegas et al., 2018). Selecting for deeper and more extensive rooting systems is a common breeding strategy. However, the correlation between root depth and drought tolerance remains unclear. The results from studies which analyzed the association of rooting depth and drought tolerance are mixed. Caradus (1981) reported that germplasm with larger leaf size and rooting system outperformed germplasm with smaller root systems. Large-leaved cultivars are often characterized by low stolon density, compared to small-leaved cultivars which have high stolon density (Widdup and Barrett, 2011). However, Barbour et al. (1996) found no significant difference between the market class of the cultivar and the performance under drought conditions. Selecting for a deeper root system is convoluted by the rooting pattern of white clover. A taproot is present for the early life of the plant (12–18 months) and afterward is replaced with a shallow nodal root system (Pederson, 1989). Caradus and Woodfield (1986) found that the cultivar with the highest proportion of root to total plant weight did not have a large proportion of taproot to total root weight, suggesting that some nodal roots were more “tap rooted.” Annicchiarico and Piano (2004) proposed an alternative in selecting for greater rooting systems by selecting for thicker stolons and found that the plants with thicker stolons had increased root dry weight.

### Drought Tolerance in White Clover

Photosynthesis is the critical process that influencing plant performance. Under drought stress, plants close stomata to conserve water, reducing photosynthesis. Stomatal conductance can be influenced by plant anatomy in long term drought (Xu and Zhou, 2008). Plant cultivars with smaller stomata have been reported to have a higher rate of gas exchange and faster stomatal response times. Faster stomatal response times can reduce the impact of drought to plants (Drake et al., 2013). This experiment gives an objective measurement of white clover cultivar transpiration rates and the critical FTSW threshold at which each cultivar begins to have permanent stomatal closure. This deepens the understanding of the expected response of cultivars to drought-like conditions.

A low FTSWc suggests that the cultivar can sustain normal transpiration for a longer period in soils with less available water implying that it would perform advantageously under short-period drought conditions, compared to cultivars with a high FTSW (Ray and Sinclair, 1997). However, He and Dijkstra (2014) found that drought stress had a stronger negative effect on plant nitrogen and phosphorus concentrations in short-term droughts compared to long-term droughts. AberHerald had the lowest FTSWc of the eighty cultivars. AberHerald is a medium leaved cultivar originating from Wales, United Kingdom. It performs well in cold environments, ensuring good stolon survival over winter. Helgadóttir et al. (2001) showed that AberHerald showed morphological adaptation to more marginal climates. AberHerald has good stolon survival and rapid stolon recovery after grazing (Rhodes and Webb, 1993). Other cultivars with a low FTSWc have high persistence under grazing (Charlton and Giddens, 1983; Widdup et al., 2006).

A high FTSWc proposes that the cultivar can perform better in long-period drought conditions as they conserve water stores by closing their stomata early (Ray and Sinclair, 1997). Tribute had the highest FTSWc value (0.52) of the eighty cultivars. Tribute is a medium-leaved cultivar bred from germplasm from New Zealand and Europe and was initially bred through a breeding program for increased drought tolerance in Australia. Woodfield et al. (2003) noted that Tribute had good drought performance in the third year of a grazing trial in Canterbury. Whilst Widdup and Barrett (2011) found that Tribute produced a greater number of stolons and stolon density than other cultivars of medium leaf size.

Generally, the cultivars released pre-1965 have a higher FTSWc than the cultivars post-1965. A high FTSWc could imply an increase in persistence in the cultivar. Figures 2A,B illustrate the relationship between average total water transpired and the average dry weights of drought and irrigated exposed plants. All three measurements follow the same trend; a peak in 1940 and decreasing to 1980, before increasing to 2010. The increase in transpiration and dry weights is supported by the study by Hoyos-Villegas et al. (2019). They found that in the same panel of 80 white clover cultivars, dry matter increased more than clover content post-1965, implying that persistence had decreased in post-1965 cultivars. Clover content, the amount of clover present in a sward, is a measurement often used to infer persistence. We could conclude that the stomatal behavior of white clover cultivars has shifted the major agronomic of persistence.

### Canopy Wilting

The underlying mechanisms of drought-tolerant phenotypes are vast. Canopy wilting is one of the first signs of drought stress caused by soil water deficits (Kunert and Vorster, 2020). In soybean, the slow-wilting phenotype was first reported in a Japanese landrace (Sloane et al., 1990) and the development of slow-wilting genotypes has enabled selection for breeding (Sinclair et al., 2010). The development of genotypes with delayed canopy wilting phenotypes have been studied thoroughly in soybean and in several plant introductions, and could lead to increased yield stability in drought conditions (Steketee et al., 2020). Simulations have suggested if the phenotype was bred into populations, yield in drought conditions could improve by >80% (Sinclair et al., 2010). However, the physiological mechanisms controlling the slow-wilting phenotype remain uncertain. Failure to understand the mechanisms will constrain breeding efforts.

Recent studies have shown some understanding of the underlying genetic architecture of the slow-wilting phenotype. Quantitative trait loci (QTLs) have been identified for canopy wilting (Abdel-Haleem et al., 2012; Ye et al., 2020) and it has been concluded that it is a polygenic trait (Charlson et al., 2009). A recent study by Kaler et al. (2017) has identified SNPs associated with canopy-wilting that are located within or close to genes with connections to transpiration or water transport. Devi et al. (2015) analyzed gene expression in the leaves of two slow-wilting accessions and showed that 944 genes were differentially expressed in one accession compared to the other. More recently, Steketee et al. (2020) used a GWAS to identify 45 marker-trait associations with canopy wilting and as a result, several new accessions were identified with the slow-wilting phenotype. Although the research into the slow-wilting phenotype has primarily been performed in soybean, other crops, such as cowpea [*Vigna unguiculata* (L.) Walp], have utilized the phenotype to identify accessions with increased drought tolerance (Pungulani, 2014).

The utilization of a slow-wilting phenotype in white clover could increase the performance under drought conditions. Although the cultivars in this study have been characterized by FTSWc under drought conditions, germplasm exploration to identify accessions with a slow-wilting phenotype is needed to accelerate breeding efforts (Barbour et al., 1996).

## Conclusion

The results from this study highlight the variable rates of stomatal closure for eighty white clover cultivars. The relationship between NTR and FTSW is consistent for all cultivars, regardless of the decade of release. Cultivars that have a significantly higher or lower FTSWc have been identified and have deepened the knowledge of the cultivar response to drought conditions, follow up studies focusing on stomatal characteristics of contrasting genotypes would be a natural next step. The white clover ISH program shows promise for increasing drought tolerance but further replicated trials are needed to assess performance. The increasing demand for cultivars to perform under extreme conditions in response to climate change requires more research into the genetic and phenotypic basis of drought traits and how these can be incorporated into breeding programs.

## Data Availability Statement

The raw data supporting the conclusions of this article will be made available by the authors, without undue reservation.

## Author Contributions

VH-V conceived the project and designed the experiments. LE and JH analyzed the data, prepared figures, and manuscript. RH and SN contributed with revisions, comments, and writing. All authors contributed to the article and approved the submitted version.

## Conflict of Interest

The authors declare that the research was conducted in the absence of any commercial or financial relationships that could be construed as a potential conflict of interest.

## Abbreviations

NTR: Normalized transpiration rate
FTSW: Fraction of transpirable soil water
VPD: Vapor pressure deficit
ISH: Interspecific hybrid.
