# Supplementary material for: Transpiration Rate of White Clover (Trifolium repens L.) Cultivars in Drying Soil
Source: Front Plant Sci. 2021 Mar 17;12:595030. doi: 10.3389/fpls.2021.595030 (PMC8010265; doi:10.3389/fpls.2021.595030)
Supplement: Supplementary file 1 [file Data_Sheet_1.docx]

**Supplementary Material:**

Supplementary table 1. The average dry weight for eighty drought exposed white clover cultivars, clustered by leaf size and decade of release. Duncan’s lettering compared the dry weights for leaf size and decade of release and the groups without a common letter are significantly different at the 5% level of probability.

| **Decade of release and leaf size** | **Dry weight average** |
| --- | --- |
| 1920 Medium | 13.58a |
| 1960 Large | 13.8a |
| 1980 Medium | 14.47ab |
| 1970 Medium | 15.04abc |
| 1990 Large | 15.17abc |
| 2000 Medium | 15.41abc |
| 1990 Medium | 15.63abc |
| 1980 Large | 15.7abcd |
| 1960 Medium | 15.75abcde |
| 1970 Large | 16.01abcde |
| 2000 Large | 16.39abcde |
| 1930 Small | 16.85abcdef |
| 1950 Medium | 17.26acdef |
| 2010 Large | 17.85acdef |
| 2010 Medium | 18.26def |
| 1930 Medium | 18.38def |
| 1950 Large | 19.23f |
| 1940 Medium | 19.36ef |

Supplementary table 2. The average dry weight for eighty irrigated white clover cultivars, clustered by leaf size and decade of release. Duncan’s lettering compared the dry weights for leaf size and decade of release and the groups without a common letter are significantly different at the 5% level of probability.

| **Decade of release and leaf size** | **Dry weight average** |
| --- | --- |
| 1920 Medium | 17.75a |
| 1960 Large | 20.23ab |
| 1970 Medium | 20.36abc |
| 1980 Medium | 20.73abc |
| 2000 Medium | 21.37abc |
| 1990 Medium | 21.39abcd |
| 1960 Medium | 21.52abcde |
| 2000 Large | 22.65abcde |
| 1970 Large | 22.95abcde |
| 1980 Large | 23.22abcde |
| 1930 Medium | 23.52abcde |
| 1990 Large | 23.63abcde |
| 2010 Medium | 24.08bde |
| 1950 Large | 24.63bcde |
| 1950 Medium | 25.71be |
| 1940 Medium | 25.86bde |
| 2010 Large | 26.05be |
| 1930 Small | 26.28bcde |

Supplementary table 3. The estimated linear spline trend: Inflection point = *a* + *b* * Time + *c* * Time_2, estimates and standard error (SE) of estimates and significance levels (*P*-values) of the estimates.

| **Parameter** | **Estimate** | **SE** | **P-value** |
| --- | --- | --- | --- |
| *a* | 0.2311 | 0.0464 | *< 0.001* |
| *b* (= increasing rate per decade till 1960) | 0.0329 | 0.0133 | *0.016* |
| *c* | − 0.0636 | 0.0199 | *0.002* |
| *b* + *c* (absolute value = decreasing rate per decade after 1960) | − 0.0307 | 0.0089 | *0.001* |
